# *N*′-[(2*E*)-3-Phenyl­prop-2-eno­yl]benzo­hydrazide

**DOI:** 10.1107/S1600536809048156

**Published:** 2009-11-18

**Authors:** Samir A. Carvalho, Edson F. da Silva, Edward R. T. Tiekink, James L. Wardell, Solange M. S. V. Wardell

**Affiliations:** a Instituto de Química, Universidade Federal do Rio de Janeiro, 21949-900, Rio de Janeiro, RJ, Brazil; b Departamento de Síntese Orgánica, Instituto de Tecnologia em Fármacos FIOCRUZ, Manguinhos, Rua Sizenando Nabuco 100, Manguinhos, 21041-250, Rio de Janeiro, RJ, Brazil; c Department of Chemistry, University of Malaya, 50603 Kuala Lumpur, Malaysia; d Centro de Desenvolvimento Tecnológico em Saúde (CDTS), Fundação Oswaldo Cruz (FIOCRUZ), Casa Amarela, Campus de Manguinhos, Av. Brasil 4365, 21040-900, Rio de Janeiro, RJ, Brazil; e CHEMSOL, 1 Harcourt Road, Aberdeen AB15 5NY, Scotland

## Abstract

In the title compound, C_16_H_14_N_2_O_2_, the conformation about the C=C bond is *E*, and the two amide groups are effectively orthogonal [the C—N—N—C torsion angle is 104.5 (2)°]. In the crystal structure, the amide groups groups associate *via* N–H⋯O hydrogen bonding, forming supra­molecular tapes with undulating topology along the *c*-axis direction.

## Related literature

For the biological activity of *trans*-cinnamic acid derivatives, see: Bezerra *et al.* (2006 ▶); Chung & Shin (2007 ▶); Naz *et al.* (2006 ▶); Rastogi *et al.* (1998 ▶); Reddy *et al.* (1995 ▶). For recent studies directed towards developing drugs for the treatment of tuberculosis, see: Carvalho *et al.* (2008 ▶).
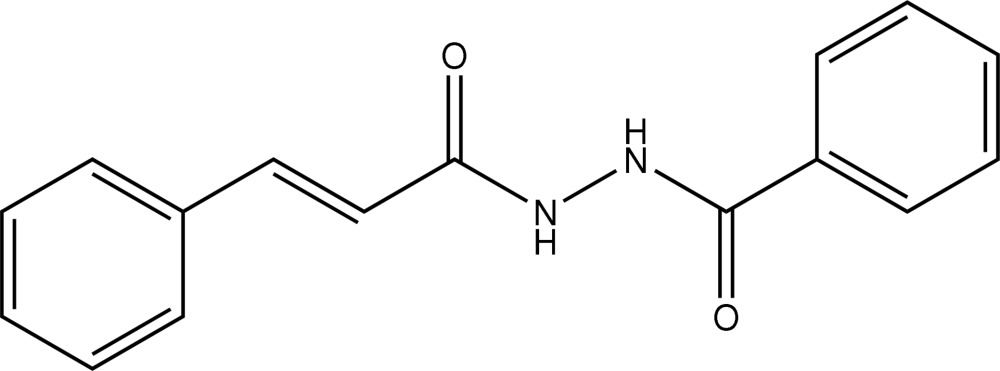


## Experimental

### 

#### Crystal data

C_16_H_14_N_2_O_2_
*M* *_r_* = 266.29
Monoclinic, 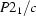

*a* = 15.9696 (7) Å
*b* = 10.4563 (5) Å
*c* = 8.3162 (2) Å
β = 102.072 (3)°
*V* = 1357.95 (9) Å^3^
*Z* = 4
Mo *K*α radiation
μ = 0.09 mm^−1^
*T* = 120 K
0.48 × 0.20 × 0.08 mm

#### Data collection

Nonius KappaCCD area-detector diffractometer
Absorption correction: multi-scan (*SADABS*; Sheldrick, 2003 ▶) *T*_min_ = 0.636, *T*_max_ = 0.746
17862 measured reflections
3110 independent reflections
2010 reflections with *I* > 2σ(*I*)
*R*_int_ = 0.086

#### Refinement

*R*[*F* ^2^ > 2σ(*F* ^2^)] = 0.053
*wR*(*F* ^2^) = 0.172
*S* = 1.10
3110 reflections
187 parameters
H atoms treated by a mixture of independent and constrained refinement
Δρ_max_ = 0.45 e Å^−3^
Δρ_min_ = −0.42 e Å^−3^

### 

Data collection: *COLLECT* (Hooft, 1998 ▶); cell refinement: *DENZO* (Otwinowski & Minor, 1997 ▶) and *COLLECT*; data reduction: *DENZO* and *COLLECT*; program(s) used to solve structure: *SHELXS97* (Sheldrick, 2008 ▶); program(s) used to refine structure: *SHELXL97* (Sheldrick, 2008 ▶); molecular graphics: *DIAMOND* (Brandenburg, 2006 ▶); software used to prepare material for publication: *publCIF* (Westrip, 2009 ▶).

## Supplementary Material

Crystal structure: contains datablocks general, I. DOI: 10.1107/S1600536809048156/hg2591sup1.cif

Structure factors: contains datablocks I. DOI: 10.1107/S1600536809048156/hg2591Isup2.hkl

Additional supplementary materials:  crystallographic information; 3D view; checkCIF report

## Figures and Tables

**Table 1:** Hydrogen-bond geometry (Å, °)

*D*—H⋯*A*	*D*—H	H⋯*A*	*D*⋯*A*	*D*—H⋯*A*
N1—H*N*1⋯O1^i^	0.89 (2)	1.95 (2)	2.827 (2)	168 (2)
N2—H*N*2⋯O2^ii^	0.86 (2)	2.01 (2)	2.852 (2)	168 (2)

Symmetry codes: (i) 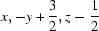; (ii) 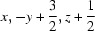.

**Fig. 1.**
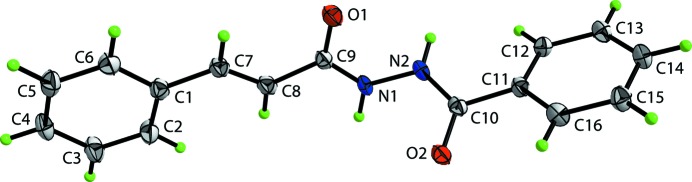
Molecular structure of (I) showing the atom-labelling scheme and displacement ellipsoids at the 50% probability level.

**Fig. 2.**
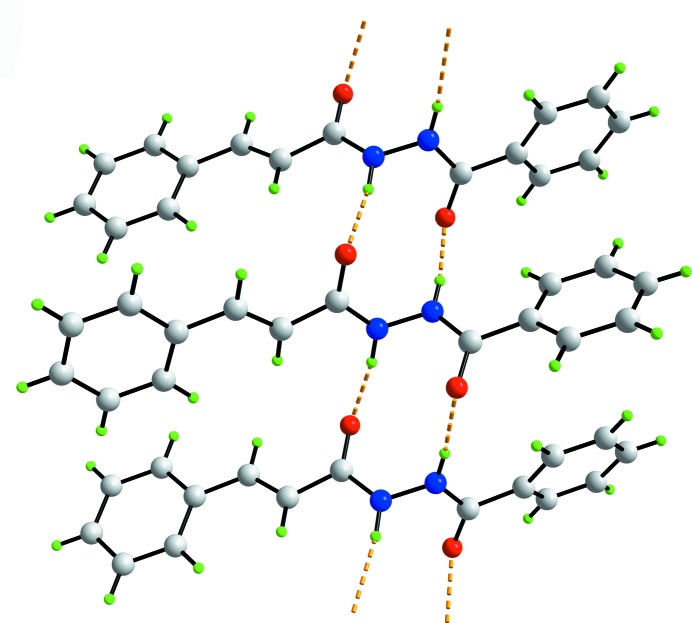
Supramolecular tape with undulating topology in (I). The N–H···O hydrogen bonding is shown as orange dashed lines

## supplementary crystallographic information

### Comment

Tuberculosis (TB) remains among the world's great public health challenges. Worldwide resurgence of TB is due to two major problems: the AIDS epidemic, which started in the mid-1980's, and the outbreak of multi-drug resistant (MDR) TB. The deadly combination of TB and HIV has led to a quadrupling of TB cases in several African and Asian countries [http://www.who.int/tdr/diseases/tb/default.htm]. MDR-TB, defined as resistance to at least isoniazid and rifamycin, two current first-line drugs, has increased morbidity and mortality with an overall increase in health care costs. The first-line treatment has some disadvantages such as important side-effects and weak sterility problems, and must be administered for 6–9 months. When standard treatments fail, second-line TB drugs are used, but these drugs have a far lower efficacy and require even longer administration periods (18–24 months) with higher cost (US $2500–3000 per treatment), higher rates of adverse effects, and low cure rates (around 60%). It is estimated that 4% of all worldwide TB patients are resistant to at least one of the current first-line drugs. TB is responsible for 20% of all deaths in adults, and each year there are about nine million new cases, of which 15% are children, and two million of deaths, of which 450.000 are children. Due to the increase of MDR-TB and AIDS cases worldwide and the lack of new drugs, there is an urgent need for new drugs to fight this disease. In our continuing research for new potent and anti-malarial agents, we reported on a new class of isonicotinic and benzoic acid *N*'-(3-phenyl-acryloyl)-hydrazide derivatives as attractive anti-tubercular agents (Carvalho *et al.*, 2008) and now report the structure of *N'*-[(*2E*)-3-phenylprop-2-enoyl]benzohydrazide, (I). The choice of *trans*-cinnamic acid derivatives in this study follows on from earlier reports of their significant biological activities (Bezerra *et al.*, 2006; Chung & Shin, 2007; Naz *et al.*, 2006; Rastogi *et al.*, 1998; Reddy *et al.*, 1995).

In (I), the conformation about the C7═C8 bond is *E*, Fig. 1. There is significant twisting in the molecule, in particular about about the central N1–N2 bond as seen in the value of the C9/N1/N2/C10 torsion angle of 104.5 (2) °. This has the consequence that the amide groups are effectively orthogonal to each other, a feature that facilitates the formation of N–H···O hydrogen bond leading to the formation of undulating supramolecular tapes in the *c* direction, Fig. 2 and Table 1.

### Experimental

4-Nitrophenyl (2E)-3-phenyl-2-propenoate (2.0 g), prepared by successive treatments of *trans*-cinnamic acid with thionyl chloride and 4-nitrophenol, was added to a solution of PhCONHNH_2_ (1.1 equiv.) in pyridine (40 ml). After refluxing the reaction mixture for 6 h, the excess of pyridine was removed under vacuum and water (20 ml) was added. The precipitate was filtered under vacuum, and washed with water to furnish (I) in 78% yield. The crystals used in the structure determination were grown from ethanol solution, m. pt. 482–483 K. ^1^H NMR (500.00 MHz, DMSO-d6) δ: 6.78 (1*H*, *d*, *J* = 16.0 Hz, Ph—CH), 7.42 (3*H*, *m*, aryl-H), 7.52 (2*H*, *m*, aryl-H), 7.60 (1*H*, *d*, *J* = 16.0 Hz, CHCO), 7.63 (3*H*, *m*, aryl-H), 7.93 (2*H*, *d*, J = 7.5 Hz, aryl-H, 10.25 (1*H*, *s*, NH), 10.56 (1*H*, *s*, NH) p.p.m. ^13^C NMR (125 MHz, DMSO-d6) δ: 165.48, 164.45, 140.34, 134.59, 132.45, 131.92, 129.91, 129.07, 127.78, 121.52, 119.45 p.p.m.

### Refinement

The C-bound H atoms were geometrically placed (C–H = 0.95 Å) and refined as riding with *U_iso_*(H) = 1.*2U_eq_*(C). The positions of the amide-N H atoms were refined with *U_iso_*(H) = 1.*2U_eq_*(N), see Table 1 for distances.

### Crystal data

**Table:**

C_16_H_14_N_2_O_2_	*F*(000) = 560
*M**_r_* = 266.29	*D*_x_ = 1.303 Mg m^−^^3^
Monoclinic, *P*2_1_/*c*	Mo *K*α radiation, λ = 0.71073 Å
Hall symbol: -P 2ybc	Cell parameters from 6527 reflections
*a* = 15.9696 (7) Å	θ = 2.9–27.5°
*b* = 10.4563 (5) Å	µ = 0.09 mm^−^^1^
*c* = 8.3162 (2) Å	*T* = 120 K
β = 102.072 (3)°	Block, light-red
*V* = 1357.95 (9) Å^3^	0.48 × 0.20 × 0.08 mm
*Z* = 4	

### Data collection

**Table:**

Nonius KappaCCD area-detector diffractometer	3110 independent reflections
Radiation source: Enraf–Nonius FR591 rotating anode	2010 reflections with *I* > 2σ(*I*)
10 cm confocal mirrors	*R*_int_ = 0.086
Detector resolution: 9.091 pixels mm^-1^	θ_max_ = 27.5°, θ_min_ = 3.2°
φ and ω scans	*h* = −20→20
Absorption correction: multi-scan (*SADABS*; Sheldrick, 2003)	*k* = −13→13
*T*_min_ = 0.636, *T*_max_ = 0.746	*l* = −9→10
17862 measured reflections	

### Refinement

**Table:**

Refinement on *F*^2^	Primary atom site location: structure-invariant direct methods
Least-squares matrix: full	Secondary atom site location: difference Fourier map
*R*[*F*^2^ > 2σ(*F*^2^)] = 0.053	Hydrogen site location: inferred from neighbouring sites
*wR*(*F*^2^) = 0.172	H atoms treated by a mixture of independent and constrained refinement
*S* = 1.10	*w* = 1/[σ^2^(*F*_o_^2^) + (0.0894*P*)^2^] where *P* = (*F*_o_^2^ + 2*F*_c_^2^)/3
3110 reflections	(Δ/σ)_max_ < 0.001
187 parameters	Δρ_max_ = 0.45 e Å^−^^3^
0 restraints	Δρ_min_ = −0.42 e Å^−^^3^

### Special details

**Table:**

Geometry. All s.u.'s (except the s.u. in the dihedral angle between two l.s. planes) are estimated using the full covariance matrix. The cell s.u.'s are taken into account individually in the estimation of s.u.'s in distances, angles and torsion angles; correlations between s.u.'s in cell parameters are only used when they are defined by crystal symmetry. An approximate (isotropic) treatment of cell s.u.'s is used for estimating s.u.'s involving l.s. planes.
Refinement. Refinement of *F*^2^ against ALL reflections. The weighted *R*-factor *wR* and goodness of fit *S* are based on *F*^2^, conventional *R*-factors *R* are based on *F*, with *F* set to zero for negative *F*^2^. The threshold expression of *F*^2^ > 2σ(*F*^2^) is used only for calculating *R*-factors(gt) *etc*. and is not relevant to the choice of reflections for refinement. *R*-factors based on *F*^2^ are statistically about twice as large as those based on *F*, and *R*- factors based on ALL data will be even larger.

### Fractional atomic coordinates and isotropic or equivalent isotropic displacement parameters (Å^2^)

**Table:**

	*x*	*y*	*z*	*U*_iso_*/*U*_eq_	
O1	0.20786 (9)	0.62832 (14)	0.42536 (16)	0.0272 (4)	
O2	0.36866 (9)	0.61547 (14)	0.11169 (15)	0.0249 (4)	
N1	0.25365 (10)	0.74825 (18)	0.2337 (2)	0.0239 (4)	
HN1	0.2442 (14)	0.779 (2)	0.132 (3)	0.029*	
N2	0.33940 (11)	0.73943 (18)	0.3148 (2)	0.0248 (4)	
HN2	0.3532 (14)	0.774 (2)	0.410 (3)	0.030*	
C1	−0.04530 (13)	0.6129 (2)	0.1175 (2)	0.0233 (5)	
C2	−0.07212 (14)	0.6812 (2)	−0.0287 (3)	0.0305 (5)	
H2	−0.0322	0.7330	−0.0692	0.037*	
C3	−0.15601 (14)	0.6745 (2)	−0.1150 (3)	0.0328 (6)	
H3	−0.1733	0.7219	−0.2138	0.039*	
C4	−0.21495 (13)	0.5991 (2)	−0.0583 (3)	0.0291 (5)	
H4	−0.2725	0.5948	−0.1177	0.035*	
C5	−0.18951 (14)	0.5302 (2)	0.0851 (3)	0.0301 (5)	
H5	−0.2297	0.4782	0.1244	0.036*	
C6	−0.10529 (13)	0.5366 (2)	0.1725 (2)	0.0264 (5)	
H6	−0.0884	0.4885	0.2709	0.032*	
C7	0.04268 (13)	0.6196 (2)	0.2143 (2)	0.0236 (5)	
H7	0.0550	0.5693	0.3115	0.028*	
C8	0.10735 (12)	0.6886 (2)	0.1809 (2)	0.0236 (5)	
H8	0.0983	0.7412	0.0857	0.028*	
C9	0.19296 (12)	0.6835 (2)	0.2911 (2)	0.0211 (4)	
C10	0.39296 (12)	0.66689 (19)	0.2472 (2)	0.0203 (5)	
C11	0.48306 (12)	0.65355 (19)	0.3422 (2)	0.0208 (5)	
C12	0.52237 (13)	0.7406 (2)	0.4602 (2)	0.0226 (5)	
H12	0.4913	0.8126	0.4859	0.027*	
C13	0.60701 (13)	0.7227 (2)	0.5409 (2)	0.0272 (5)	
H13	0.6338	0.7826	0.6213	0.033*	
C14	0.65253 (14)	0.6170 (2)	0.5038 (2)	0.0280 (5)	
H14	0.7105	0.6050	0.5582	0.034*	
C15	0.61300 (13)	0.5296 (2)	0.3873 (2)	0.0278 (5)	
H15	0.6438	0.4569	0.3631	0.033*	
C16	0.52891 (13)	0.5473 (2)	0.3060 (2)	0.0255 (5)	
H16	0.5024	0.4873	0.2255	0.031*	

### Atomic displacement parameters (Å^2^)

**Table:**

	*U*^11^	*U*^22^	*U*^33^	*U*^12^	*U*^13^	*U*^23^
O1	0.0249 (8)	0.0309 (9)	0.0232 (7)	0.0004 (6)	−0.0008 (6)	0.0013 (6)
O2	0.0223 (8)	0.0286 (9)	0.0214 (7)	−0.0042 (6)	−0.0009 (5)	−0.0019 (6)
N1	0.0140 (9)	0.0337 (11)	0.0212 (8)	−0.0004 (8)	−0.0030 (6)	0.0011 (8)
N2	0.0164 (9)	0.0348 (11)	0.0202 (8)	−0.0005 (8)	−0.0033 (6)	−0.0027 (8)
C1	0.0197 (11)	0.0247 (12)	0.0245 (10)	0.0021 (9)	0.0024 (8)	−0.0015 (8)
C2	0.0213 (11)	0.0354 (13)	0.0335 (11)	−0.0057 (10)	0.0026 (9)	0.0068 (10)
C3	0.0247 (12)	0.0371 (14)	0.0323 (12)	−0.0049 (10)	−0.0038 (9)	0.0084 (10)
C4	0.0174 (11)	0.0292 (13)	0.0369 (12)	0.0000 (9)	−0.0029 (9)	−0.0022 (9)
C5	0.0244 (12)	0.0288 (12)	0.0368 (12)	−0.0087 (10)	0.0056 (9)	−0.0024 (10)
C6	0.0249 (11)	0.0270 (12)	0.0264 (10)	−0.0020 (9)	0.0031 (8)	0.0006 (9)
C7	0.0216 (11)	0.0270 (12)	0.0212 (10)	0.0030 (9)	0.0025 (8)	−0.0012 (8)
C8	0.0218 (11)	0.0262 (11)	0.0209 (10)	0.0041 (9)	0.0004 (8)	−0.0011 (8)
C9	0.0183 (10)	0.0222 (11)	0.0213 (10)	0.0015 (9)	0.0005 (7)	−0.0048 (8)
C10	0.0193 (11)	0.0218 (11)	0.0188 (10)	−0.0031 (8)	0.0014 (7)	0.0041 (8)
C11	0.0181 (10)	0.0231 (11)	0.0204 (10)	−0.0020 (8)	0.0019 (7)	0.0043 (8)
C12	0.0189 (10)	0.0268 (12)	0.0218 (9)	−0.0014 (9)	0.0037 (7)	−0.0001 (8)
C13	0.0202 (11)	0.0328 (13)	0.0264 (10)	−0.0028 (9)	−0.0002 (8)	−0.0034 (9)
C14	0.0181 (10)	0.0357 (13)	0.0274 (11)	0.0019 (9)	−0.0017 (8)	0.0010 (9)
C15	0.0249 (12)	0.0277 (12)	0.0298 (11)	0.0069 (9)	0.0034 (8)	0.0038 (9)
C16	0.0253 (12)	0.0252 (12)	0.0242 (10)	−0.0008 (9)	0.0015 (8)	0.0003 (8)

### Geometric parameters (Å, °)

**Table:**

O1—C9	1.235 (2)	C6—H6	0.9500
O2—C10	1.235 (2)	C7—C8	1.336 (3)
N1—C9	1.348 (3)	C7—H7	0.9500
N1—N2	1.397 (2)	C8—C9	1.479 (3)
N1—HN1	0.89 (2)	C8—H8	0.9500
N2—C10	1.351 (3)	C10—C11	1.496 (3)
N2—HN2	0.86 (2)	C11—C12	1.388 (3)
C1—C6	1.395 (3)	C11—C16	1.398 (3)
C1—C2	1.398 (3)	C12—C13	1.390 (3)
C1—C7	1.467 (3)	C12—H12	0.9500
C2—C3	1.383 (3)	C13—C14	1.392 (3)
C2—H2	0.9500	C13—H13	0.9500
C3—C4	1.384 (3)	C14—C15	1.384 (3)
C3—H3	0.9500	C14—H14	0.9500
C4—C5	1.379 (3)	C15—C16	1.384 (3)
C4—H4	0.9500	C15—H15	0.9500
C5—C6	1.390 (3)	C16—H16	0.9500
C5—H5	0.9500		
			
C9—N1—N2	120.03 (17)	C7—C8—C9	120.44 (18)
C9—N1—HN1	121.9 (14)	C7—C8—H8	119.8
N2—N1—HN1	116.0 (14)	C9—C8—H8	119.8
C10—N2—N1	118.56 (16)	O1—C9—N1	122.52 (17)
C10—N2—HN2	124.2 (16)	O1—C9—C8	123.72 (18)
N1—N2—HN2	117.0 (15)	N1—C9—C8	113.74 (17)
C6—C1—C2	118.08 (18)	O2—C10—N2	121.24 (17)
C6—C1—C7	119.48 (18)	O2—C10—C11	121.69 (17)
C2—C1—C7	122.44 (19)	N2—C10—C11	117.06 (16)
C3—C2—C1	120.8 (2)	C12—C11—C16	119.57 (18)
C3—C2—H2	119.6	C12—C11—C10	123.71 (18)
C1—C2—H2	119.6	C16—C11—C10	116.71 (18)
C2—C3—C4	120.41 (19)	C11—C12—C13	120.21 (19)
C2—C3—H3	119.8	C11—C12—H12	119.9
C4—C3—H3	119.8	C13—C12—H12	119.9
C5—C4—C3	119.58 (19)	C12—C13—C14	119.99 (19)
C5—C4—H4	120.2	C12—C13—H13	120.0
C3—C4—H4	120.2	C14—C13—H13	120.0
C4—C5—C6	120.3 (2)	C15—C14—C13	119.76 (19)
C4—C5—H5	119.9	C15—C14—H14	120.1
C6—C5—H5	119.9	C13—C14—H14	120.1
C5—C6—C1	120.80 (19)	C14—C15—C16	120.5 (2)
C5—C6—H6	119.6	C14—C15—H15	119.8
C1—C6—H6	119.6	C16—C15—H15	119.8
C8—C7—C1	127.24 (19)	C15—C16—C11	119.97 (19)
C8—C7—H7	116.4	C15—C16—H16	120.0
C1—C7—H7	116.4	C11—C16—H16	120.0
			
C9—N1—N2—C10	104.5 (2)	C7—C8—C9—N1	173.98 (19)
C6—C1—C2—C3	0.7 (3)	N1—N2—C10—O2	4.4 (3)
C7—C1—C2—C3	−178.7 (2)	N1—N2—C10—C11	−176.55 (16)
C1—C2—C3—C4	−0.3 (4)	O2—C10—C11—C12	156.22 (19)
C2—C3—C4—C5	−0.1 (3)	N2—C10—C11—C12	−22.8 (3)
C3—C4—C5—C6	0.1 (3)	O2—C10—C11—C16	−23.0 (3)
C4—C5—C6—C1	0.3 (3)	N2—C10—C11—C16	158.00 (18)
C2—C1—C6—C5	−0.7 (3)	C16—C11—C12—C13	0.5 (3)
C7—C1—C6—C5	178.8 (2)	C10—C11—C12—C13	−178.68 (17)
C6—C1—C7—C8	−179.0 (2)	C11—C12—C13—C14	−0.2 (3)
C2—C1—C7—C8	0.4 (3)	C12—C13—C14—C15	−0.5 (3)
C1—C7—C8—C9	−179.37 (19)	C13—C14—C15—C16	0.8 (3)
N2—N1—C9—O1	9.3 (3)	C14—C15—C16—C11	−0.5 (3)
N2—N1—C9—C8	−172.41 (17)	C12—C11—C16—C15	−0.1 (3)
C7—C8—C9—O1	−7.8 (3)	C10—C11—C16—C15	179.11 (17)

### Hydrogen-bond geometry (Å, °)

**Table:**

*D*—H···*A*	*D*—H	H···*A*	*D*···*A*	*D*—H···*A*
N1—HN1···O1^i^	0.89 (2)	1.95 (2)	2.827 (2)	168 (2)
N2—HN2···O2^ii^	0.86 (2)	2.01 (2)	2.852 (2)	168 (2)

Symmetry codes: (i) *x*, −*y*+3/2, *z*−1/2; (ii) *x*, −*y*+3/2, *z*+1/2.
